# Exploratory Assessment of Galectin-1, -3, and -9 in Non-Small Cell Lung Cancer

**DOI:** 10.3390/cancers16061165

**Published:** 2024-03-15

**Authors:** Hayden Shuster, Avery Funkhouser, Lorie Allen, Moonseong Heo, Julie C. Martin, W. Jeffery Edenfield, Anna V. Blenda

**Affiliations:** 1 Department of Biomedical Sciences, University of South Carolina School of Medicine Greenville, Greenville, SC 29605, USA; hshuster@email.sc.edu (H.S.); averytf@email.sc.edu (A.F.); 2 Prisma Health Cancer Institute, Prisma Health, Greenville, SC 29605, USAjulie.martin@prismahealth.org (J.C.M.); jeffery.edenfield@prismahealth.org (W.J.E.); 3 Department of Public Health Sciences, Clemson University, Clemson, SC 29634, USA; mheo@clemson.edu; 4 Department of Medicine, University of South Carolina School of Medicine Greenville, Greenville, SC 29605, USA

**Keywords:** non-small cell lung cancer, NSCLC, galectins, ELISA, treatment, metastasis, overall survival, prognosis

## Abstract

**Simple Summary:**

Galectins play a role in cancer angiogenesis, growth, and metastasis. Our pilot study aims to explore the role of galectins in non-small cell lung cancer (NSCLC) by assessing their correlation with treatment, metastasis, and survival. Galectin-1, -3, and -9 levels were measured in the sera of NSCLC patients using ELISA and analyzed by histology, previous treatment, metastasis, and overall survival (OS). Galectin-3 level was elevated in patients with adenocarcinoma compared to squamous cell carcinoma patients and lower in patients exposed to chemotherapy. Galectin-1 levels were significantly lower in patients with previous metastasis but did not correlate with future metastasis. Abnormal galectin-1 levels were significantly correlated with decreased OS; however, a loss of significance occurred when abnormal galectin-1 levels were examined in multivariable analysis. These preliminary findings may hold clinical importance, given the current development of galectin-3 inhibitors for NSCLC, and they underscore the potential of galectin-1 as a prognostic biomarker.

**Abstract:**

Galectins play a pivotal role in lung cancer oncogenic pathways, influencing apoptosis, angiogenesis, and tumor metastasis. Biomarkers that diagnose, prognose, and guide cancer treatment are crucial, with galectins having the biomarker potential for non-small cell lung cancer (NSCLC). Using enzyme-linked immunosorbent assay (ELISA), we assessed serum galectin-1, -3, and -9 levels in NSCLC patients. A retrospective chart review was performed to examine patient demographics, cancer stage, tumor biology, cancer treatment, and patient outcomes. Galectin levels were then compared across these factors. In this exploratory analysis, galectin-3 levels were significantly lower in patients with squamous cell lung cancer (*p* = 0.0019) and in patients exposed to chemotherapy (*p* = 0.0375). Galectin-1 levels were significantly lower in patients with previous metastasis but had no correlation with future metastasis. Abnormal galectin-1 levels were significantly correlated with decreased overall survival (OS) in NSCLC (*p* = 0.0357) and specifically in patients with surgically resectable NSCLC (*p* = 0.0112). However, abnormal galectin-1 levels were not found to correlate with decreased OS in multivariable analysis (*p* = 0.0513). These findings may have clinical implications as galectin-3 inhibitors are in trials for NSCLC. Additionally, they suggest that galectin-1 has potential as a prognostic marker for surgically resectable NSCLC.

## 1. Introduction

Despite a decrease in the incidence and mortality of lung cancer over the past two decades, and even with increasing therapeutic options, the morbidity and mortality remain substantial [1]. Lung cancer is the second most diagnosed cancer in the United States and was estimated to cause 127,070 deaths in 2023 [2]. The diagnosis carries a significant impact on patients, their caregivers, and their quality of life [3]. Additionally, the economic burden of lung cancer was estimated to be USD 12.12 billion in 2010 and is likely to rise with the advent of newer treatments such as immunotherapy [4]. Thus, optimizing resources with improved diagnostic, prognostic, and therapeutic options are critical.

Lung cancer is broadly characterized into small cell lung cancer (SCLC) and non-small cell lung cancer (NSCLC), with NSCLC constituting 85% of cases. NSCLC contains multiple subtypes, with the most common histology being adenocarcinoma, squamous cell carcinoma, and large cell carcinoma, in that order [5]. Morbidity and mortality vary between types of lung cancer and stage of diagnosis. Despite its high initial response rate to chemotherapy, SCLC exhibits a median survival time of 18 months for limited disease and 9 months for extensive disease [6]. At the same time, NSCLC demonstrates a better prognosis depending on the stage, with a 5-year survival rate of 68.4% for stage I and 5.8% for stage IV [7].

Galectins are a subfamily of lectin proteins capable of binding β-galactoside glycoconjugates through conserved carbohydrate recognition domains (CRD) [8]. Galectins are categorized by structure into three subgroups. Galectin-1, -2, -5, -7, -10, -11, -13, -14, and -15 make up the prototypical galectins that contain a sole CRD and form non-covalently linked dimers [9]. Galectin-3 is the sole member of the chimeric subgroup of galectins and has profibrotic and proinflammatory modulating macrophage activity in various tissue types [10]. The final subgroup consists of tandem-repeat galectins that have two distinct CRDs and includes galectin-4, -6, -8, -9, and -12 [11,12]. Due to the variety of structures, galectins can bind a wide range of ligands in multiple cellular pathways, including adhesion, aggregation, angiogenesis, apoptosis, autophagy, growth, and metastasis [13,14,15].

The biochemical functions of galectins are of interest in oncology with galectin-1, -3, -4, -7, -8, and -9 being the most extensively studied in lung cancer [16]. Galectin-1 has been shown to promote lung cancer metastasis by increasing levels of Notch and its ligand, Jagged2, while enhancing AKT activation, promoting tumorigenesis and invasiveness, and is associated with a poor prognosis in lung adenocarcinoma [17,18]. The knockdown of galectin-3 has been shown to decrease tumor initiation, aggressiveness, and chemoresistance to cisplatin and paclitaxel in lung adenocarcinoma. Additionally, it is involved with β-catenin, which may contribute to the maintenance of lung cancer stem cells [19]. Finally, galectin-9 on tumor-infiltrating lymphocytes (TIL) in NSCLC has been shown to correlate with T cell immunoglobin and mucin domain containing protein 3 (TIM-3,) as well as the NSCLC drug targets PD-1 and PD-L1 upregulating IFNβ and γ to decrease lung cancer apoptosis [20,21].

Due to galectins’ role in lung cancer, there have been multiple studies that have examined galectin expression and levels using immunohistochemistry (IHC) and enzyme-linked immunosorbent assay (ELISA) methodology, respectively. High galectin-1 IHC expression was associated with poor clinical outcome in NSCLC patients [22]. Additionally, galectin-3 IHC expression is decreased in SCLC when compared to NSCLC, and studies have demonstrated that higher serum and tumor levels of galectin-3 in NSCLC are associated with lymph node metastasis and tumor recurrence [23,24,25]. Lastly, high IHC expression of galectin-9 on TIL in surgically resected SCLC correlated with improved recurrence-free survival [26].

This study aims to build upon existing research by utilizing ELISA to analyze the concentrations of galectin-1, -3, and -9 in patients’ sera across various stages of the two largest histologic subtypes of NSCLC: adenocarcinoma and squamous cell carcinoma. To our knowledge, this is the first study to retrospectively review lung cancer treatments administered prior to the assessment of galectin levels, allowing us to explore the influence of NSCLC treatment on galectin levels. Finally, the study examines galectin levels in relationship to metastasis and probes the potential prognostic ability of galectin-1 in surgically resectable NSCLC patients, irrespective of prior treatment status.

## 2. Materials and Methods

### 2.1. Patient Samples

A random and heterogeneous sample set of 87 patients was collected from a single center at the Prisma Health Cancer Institute (PHCI) biorepository in Greenville, SC, USA. The samples included both tissue and serum and were collected from 2013 to 2022. This was approved by the Institutional Review Board of Prisma Health, Committee C on 05/30/2023 under 1949871-1 Use of Patient Data from the Prisma Health Center Biorepository for Molecular and Bioinformatics Analysis. The standard operating procedures of the PHCI biorepository have been described and acknowledged in multiple previous publications [27,28,29,30]. Among the patient samples, 49 consisted of adenocarcinoma and 38 of squamous cell carcinoma. Of these samples, 33 were categorized as stage I, 15 as stage II, 18 as stage III, and 21 as stage IV lung cancer. The samples were obtained from patients having both resection or biopsy of primary tumors (n = 68) and metastatic tissue (n = 19). The metastatic samples included 14 brain tissues obtained via craniotomy, 2 lymph node biopsies, 2 chest wall biopsies, and one sample from spinal cord. Patient serum was obtained in the pre-operative setting, regardless of procedure (surgical resection or biopsy).

### 2.2. Patient Information

Patient information was collected from the PHCI database and EPIC^®^. The PHCI information included demographic (age, race, sex, and smoking status) and tumor data (tissue site, histology, grade, and TNM staging). PHCI data were supplemented by information obtained from EPIC^®^ to ensure data concordance. Discrepancies between the PHCI database and EPIC^®^ were rectified through comprehensive chart review in EPIC^®^. Several samples, procured prior to the transition to EPIC^®^ as the electronic medical record (EMR) in 2015, had missing data.

Treatment, metastasis, and survival data were collected starting on 1 August 2023. The data encompassed treatment information including surgery, radiation, chemotherapy, targeted therapy, and immunotherapy exposures before and after tissue sample collection. All treatments, including individual pharmacologic agents and surgery types, were recorded if available and are documented in the Supplemental Data File S1. Some patients had additional primary cancers and were exposed to various treatments before their diagnosis of lung cancer. Their treatment exposure was documented and is available in the Supplemental Data File S1.

Information regarding metastasis was recorded utilizing EPIC^®^. Patients were stratified as being metastatic at time of sample procurement, or as not having metastatic disease. In cases where metastasis developed during the trial, time to metastasis was calculated based on the duration in weeks between the date of tissue sample collection and the date of radiographically confirmed metastasis. Subsequent physician documentation of metastasis was verified. Metastases occurring after 1 August 2023 were not recorded. Similarly, overall survival was calculated in weeks from time of sample procurement to death or survival on 1 August 2023.

### 2.3. Galectin Profiling

The galectin levels in the patients’ sera were determined using an ELISA technology described previously and included a subset of those patients [28]. Galectin-1, -3, and -9 concentrations were obtained using ELISA kits from R&D Systems (Minneapolis, MN, USA), with each sample assayed four times.

### 2.4. Data Analysis

All statistical analyses were performed using JMP^®^ 17.2.0 software by the SAS Institute (Cary, NC, USA). The distributions of the serum galectin levels were analyzed for normality using q-q plots and histogram visual inspection. Galectin levels are reported in Log_10_ as this ensures data normality for subsequent parametric analysis. Notably, three samples did not have galectin-3 levels determined, and some tumor, treatment, metastasis, and survival data were missing, precluding analysis for those specific tissue samples. Two-group comparisons were conducted using pooled or unpooled *t*-test depending upon equal or unequal variance by Levene’s test. Multiple group comparisons were analyzed with one-way ANOVA.

Survival analyses using log-rank tests and Cox proportional-hazards models were performed to compare time to metastasis and overall survival across different galectin levels and compared to healthy control levels defined previously. In our study, patients had galectin-1 levels that were below, within, or above the normal range of 13.90–28.20 ng/mL. Galectin-3 and galectin-9 had normal range values, 2.40–15.70 ng/mL and 3.10–10.40 ng/mL, respectively, as well as high values [28]. None of the sera samples tested had low levels of galectin-3 or -9. For galectin-1, survival analysis was first performed on all three groups (low, normal, and high) and then as a combination of the below and above normal ranges, called abnormal. This combination allowed for galectin-1 to be similarly compared to galectin-3 and -9 as all three galectins would have two groups (normal and abnormal). If there were statistically significant differences, further groupings by lung cancer histology and surgical resectability were performed to assess possible differences. As our population had more surgically resectable disease compared to the general lung cancer population, such analysis could assess potential differences in those patients [31].

If significant differences were identified by log-rank test, a Cox proportional-hazards model followed to adjust for demographic factors and NSCLC stage. Statistical significance was declared when a two-sided *p*-value < 0.05. All patient outcome data are available in the Supplemental Data File S1.

## 3. Results

### 3.1. Patient Characteristics Correlations with Galectin-1, -3, and -9 Levels

Serum galectin concentrations were compared by the following demographic characteristics (Table 1): age, sex, race, ethnicity, smoking status, and body mass index (BMI). Age was measured numerically in years with the mean being 65.89. Biological sex was defined as female and male. Race included Black/African American, Hawaiian/Pacific Islander, Latino, Multi-racial, and White. Since there was only one patient each that identified as Hawaiian/Pacific Islander, Latino, and Multi-racial, they were combined and called Multicultural Cohort so that statistical analysis could be performed. Ethnicity included non-Spanish/non-Hispanic and Spanish/Hispanic. Smoking status was described as current, never, or previous. BMI was defined as underweight (<18.5), healthy (18.5–24.9), overweight (25–29.9), or obese (>29.9).

Galectin-1 levels were lower in Spanish or Hispanic patients compared to Non-Spanish, Non-Hispanic patients, *p* = 0.0195. Additionally, galectin-3 and -9 levels were higher in females than in males, *p* = 0.0377 and *p* = 0.0124, respectively. All means, standard deviations, and *p*-values of associated tests are presented in Table 1.

### 3.2. Galectin-1, -3, and -9 Levels Do Not Differ by Lung Cancer Stage

**Figure 1:**
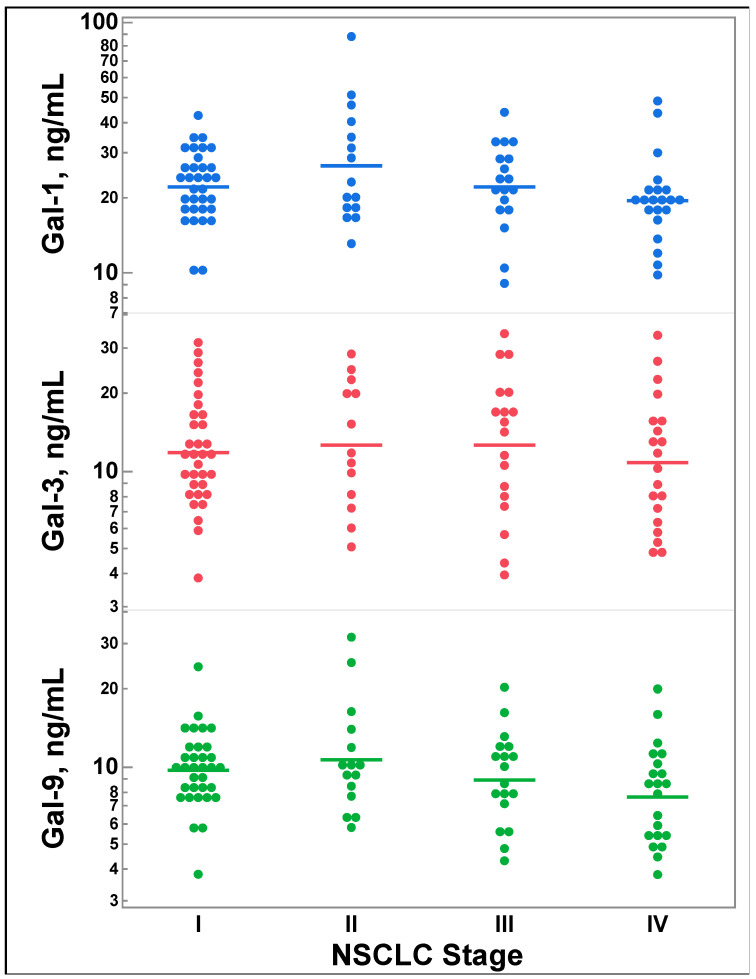
Comparison of galectin-1, -3, and -9 levels by lung cancer stage (I-IV). Multiple comparisons were performed using one-way ANOVA. Gal = galectin; NSCLC = non-small cell lung cancer.

There were no statistically significant differences between galectin-1 (*p* = 0.1275), galectin-3 (*p* = 0.8060), or galectin-9 (*p* = 0.0794) levels and NSCLC stage (Figure 1), defined as stage I, II, III, and IV, as shown in Figure 1.

### 3.3. Galectin-3 Levels Vary by Lung Cancer Histology

**Figure 2:**
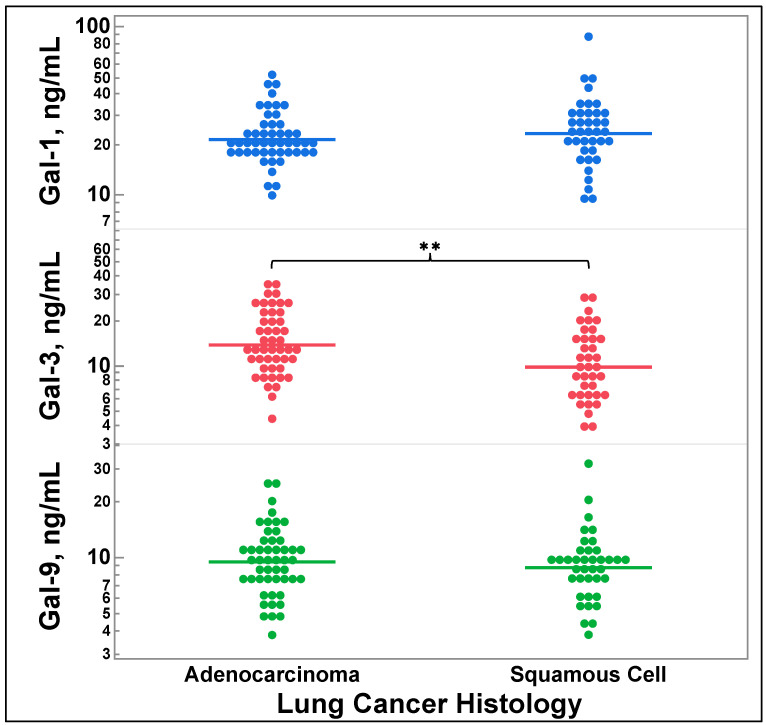
Comparison of galectin-1, -3, and -9 levels by lung cancer histology (adenocarcinoma and squamous cell carcinoma). Two group comparisons were performed by two-tailed *t*-test. ** *p* = 0.0019. Gal = galectin.

Figure 2 depicts comparisons of galectin levels by lung cancer histology. Patients with squamous cell carcinoma had decreased galectin-3 levels compared to those with adenocarcinoma (*p* = 0.0019). There were no significant differences observed for galectin-1 (*p* = 0.8264) or galectin-9 (*p* = 0.2236).

### 3.4. Galectin-1, -3, and -9 Levels and Previous Treatment Status

**Figure 3:**
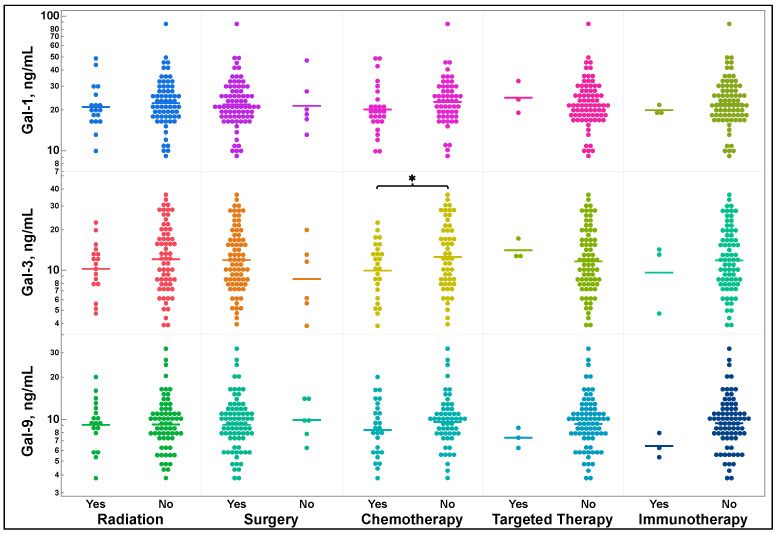
Comparison of galectin-1, -3, and -9 levels by previous lung cancer treatment type. All individual treatment agents and types of surgery received by patients are available in the Supplemental Data File S1. *P*-values, means, and standard deviations are shown in Table 2. Two group comparisons for radiation, surgery, chemotherapy, targeted therapy, and immunotherapy were made by two-tailed *t*-test. * *p* = 0.0375. Gal = galectin.

Lung cancer treatments and treatments for synchronous or metachronous cancers received by patients before sample acquisition were recorded and stratified by type as radiation, surgery, chemotherapy, targeted therapy, or immunotherapy. Subsequent treatments that patients received for their lung cancer, as well as additional or secondary cancers, such as post-operative radiation, were recorded and stratified the same way and are available in the Supplemental Data File S1. As shown in Figure 3, there were no significant differences in galectin-1, -3, or -9 levels, regardless of the previous treatments, except that those treated with chemotherapy had significantly lower levels of galectin-3 (*p* = 0.0375). Nonetheless, the trend towards lower levels of galectin-1 and galectin-9 was seen in patients exposed to chemotherapy, but it was not statistically significant (*p* = 0.0852 and 0.0940, respectively).

Table 2 presents all the associated *p*-values, means, and SDs associated with Figure 3 where two group comparisons for each treatment type were performed by two-tailed *t*-test.

### 3.5. Galectin-1, -3, and -9 Levels Do Not Correlate with Future Metastasis

**Figure 4:**
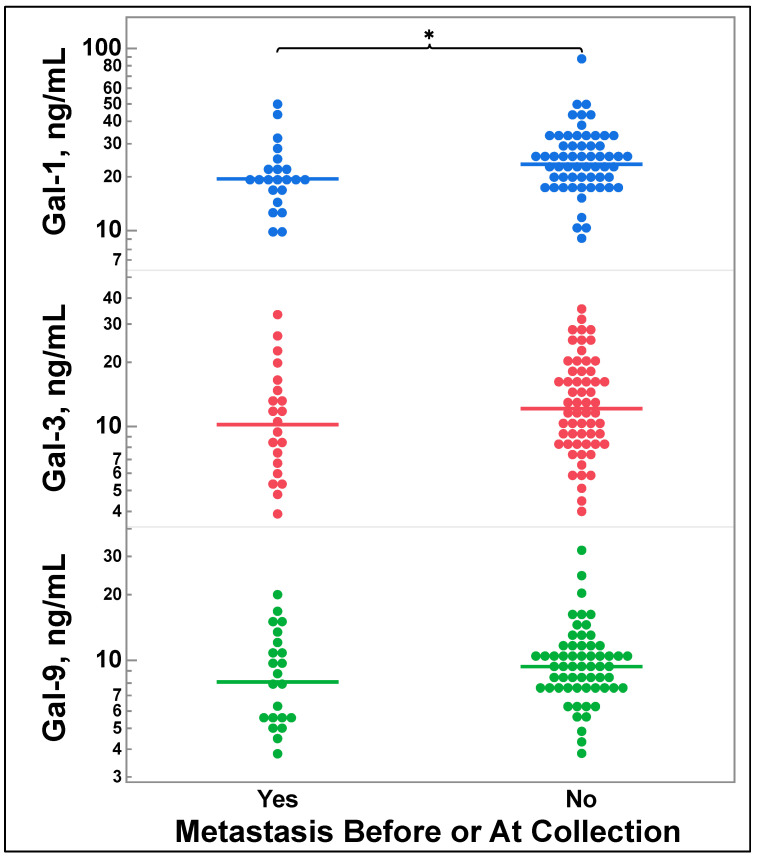
Comparison of galectin-1, -3, and -9 levels by those with metastatic disease at time of sample procurement. Two group comparisons were made by two-tailed *t*-test. * *p* = 0.0344. Gal = galectin.

Patients that had metastatic disease before or at sample acquisition had significantly lower galectin-1 levels than those without metastatic disease (*p* = 0.0344), but there was no significant difference for galectin-3 (*p* = 0.1039) or galectin-9 (*p* = 0.0582), as seen in Figure 4.

**Figure 5:**
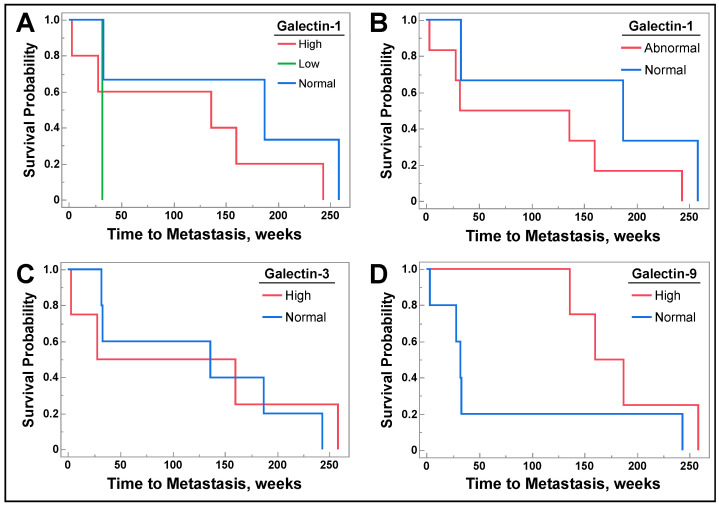
Survival probability in all graphs represents survival without development of metastatic disease in patients with stage III disease or lower. If metastasis occurred, it was recorded from date of radiologic progression with physician documentation of metastases verified. Patients with stage IV disease at lung cancer tissue procurement were excluded from this analysis. In (**A**), patients were retrospectively followed by examining the time to developing metastasis based upon serum galectin-1 level stratified as low, normal, or high. In (**B**), patients with low or high galectin-1 levels were evaluated and combined into one group, called abnormal, and the time to developing metastasis was examined. (**C**,**D**) represent levels of galectin-3 and -9, respectively, while examining time to developing metastasis by serum galectin levels. Log Rank analysis was utilized for all graphs.

However, among patients without metastatic disease at sample procurement who subsequently developed metastatic disease during follow-up, there was no correlation between serum galectin-1 level (Figure 5A, *p* = 0.3505), or when high or low galectin-1 was combined into abnormal galectin-1 level (Figure 5B, *p* = 0.3169) and development of future metastasis. Likewise, there was no correlation between galectin-3 (Figure 5C, *p* = 0.8141) or galectin-9 (Figure 5D, *p* = 0.1060) level and future metastasis.

### 3.6. Galetin-1 May Be a Prognostic Biomarker for Select Populations of NSCLC

**Figure 6:**
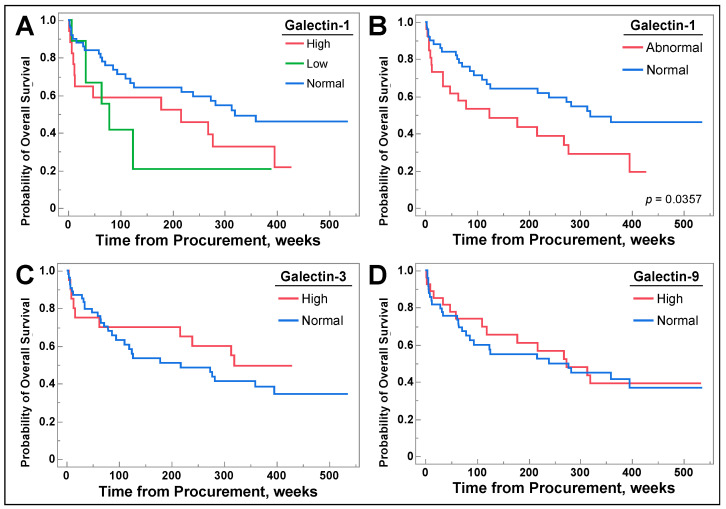
In (**A**), patients were stratified by serum galectin-1 levels, including low, normal, and high, and retrospectively followed until death or end of study duration. (**B**) combines patients with high or low galectin-1 levels into a category called abnormal, and patients with normal or abnormal galectin-1 level were followed till death or end of study duration. In (**C**,**D**), patients with normal and high galectin-3 and -9 levels, respectively, were followed until death or end of study duration. Log Rank analysis was utilized for all graphs.

Patient survival was measured in weeks from sample procurement to death or end of study duration. After OS was determined, Kaplan–Meier curves were generated to assess whether galectin-1, -3, or -9 levels correlated with OS by utilizing log rank analysis. Patients with abnormal galectin-1 (high + low) had decreased OS (Figure 6B, *p* = 0.0357). Difference in galectin-1 levels that were further delineated into high or low (Figure 6A, *p* = 0.0953) lost statistical significance. There were no differences in survival for patients with high levels of galectins-3 (Figure 6C, *p* = 0.3889) and galectin-9 (Figure 6D, *p* = 0.8047).

**Figure 7:**
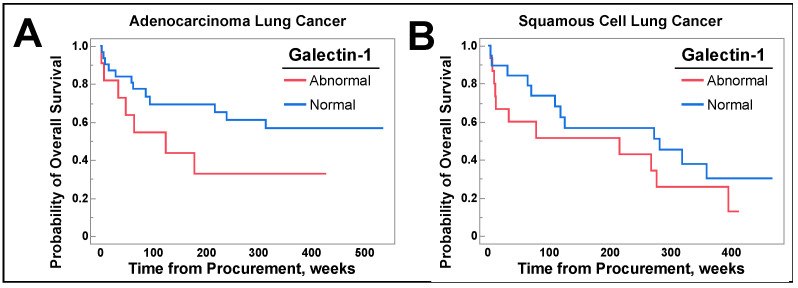
(**A**) shows patients with adenocarcinoma grouped by normal and abnormal serum galectin-1 levels, which included those with high and low levels, followed from time of lung cancer tissue collection until death or end of study duration. In (**B**), patients with squamous cell carcinoma were stratified by serum galectin-1 levels, as described above, and followed until death or end of study duration. Log Rank analysis was utilized for all graphs.

Since there was a difference in survival for patients with abnormal galectin-1 levels, we performed subsequent analysis grouped by histology and separately by surgically resectable and non-resectable disease. There were no significant differences in OS when patients were grouped by histological subtype, as seen in Figure 7.

**Figure 8:**
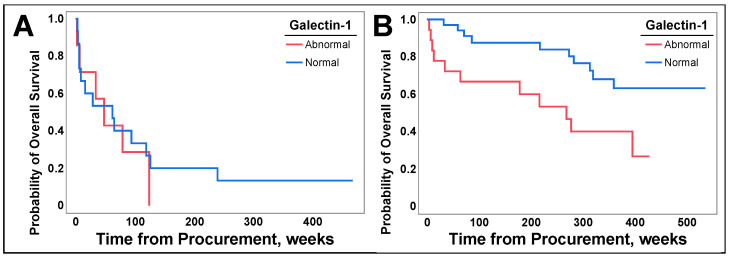
(**A**) displays patients with surgically unresectable lung cancer grouped by normal and abnormal serum galectin-1 levels, which included those with high and low levels, followed from time of lung cancer tissue collection until death or end of study duration. In (**B**), patients with resectable lung cancer were stratified by serum galectin-1 levels, as described above, and followed until death or end of study duration. Log Rank analysis was utilized for all graphs.

However, patients with surgically resectable disease and abnormal galectin-1 levels had decreased overall survival (Figure 8B, *p* = 0.0112), and there was no difference in OS observed for patients with non-resectable disease (Figure 8A, *p* = 0.5993).

Finally, a Cox proportional hazards model that included the demographic factors in Table 1 and lung cancer stage showed that abnormal galectin-1 levels were not significantly correlated with OS, with a hazard ratio of 2.928 (95% CI of 0.9940 to 8.625), yet it was borderline significant with *p* = 0.0513. All hazard ratios and 95% CI values for the Cox proportional hazards model are available in the Supplemental Data File S2.

## 4. Discussion

### 4.1. Findings

The analyzed demographics were chosen due to their associations with lung cancer [32]. Galectin-1 levels were lower in patients who identified as Spanish/Hispanic. Additionally, galectin-3 and -9 levels were higher in females compared to males. Previous studies have demonstrated increased levels of galectin-3 in females; however, this observation was made in a population at risk for stroke [33]. To the best of our knowledge, galectin-9 levels have not been demonstrated to be increased in females within the cancer population.

In our study, there were no differences between galectin-1, -3, or -9 levels and NSCLC stage. A recent study revealed a correlation between galectin-3 expression and squamous cell lung cancer stage. This finding, which is contrary to our study, might be attributed to the difference in methodologies; we employed ELISA for quantifying galectin-3 levels, whereas IHC was used to assess galectin-3 expression in the previous study [34]. Clinically, the Eighth Edition of TNM classification is utilized for staging NSCLC, but we utilized simple stage classification defined as I, II, III, and IV since we had only one patient with stage IIIC disease and two patients with stage IIIB disease [35,36]. The simple stage classification allowed for more homogenous comparisons across the disease spectrum but may be contributing to the lack of observed difference for galectin-3 compared to the above-mentioned study. Thus, further studies are necessary to assess galectin levels as they relate to lung cancer stage in squamous cell carcinoma.

Figure 2 depicts the decreased galectin-3 levels measured in the serum of patients with squamous cell carcinoma versus those with adenocarcinoma. Previous studies have identified a difference in galectin-3 levels between NSCLC and SCLC utilizing IHC [23]. However, this is the first study to identify differences in galectin-3 levels in NSCLC’s two most common subtypes. Notwithstanding, the mechanism by which galectin-3 levels vary among histological subtype of lung cancer remains unknown and is a limitation of this exploratory study.

The differences in galectin-3 levels among patients with squamous cell carcinoma and adenocarcinoma may have future clinical treatment implications as galectin-3 inhibitors are currently in development [37,38]. It is possible that response rates to galectin-3 inhibitors may differ depending on patients’ galectin-3 levels, with our data suggesting that adenocarcinoma may be the most likely to respond. We conjecture that it may be similar to how PDL1 positivity predicts response to pembrolizumab therapy (albeit imperfectly), as previous trials have utilized different cutoffs for PD-L1 positivity in NSCLC [39,40,41]. This should be further examined in clinical trials and may influence future trial design for galectin-3 inhibitors, including their subgroup analysis, as squamous cell carcinoma and non-squamous cell carcinoma (including adenocarcinoma) are currently treated with different regimens [42,43,44,45].

Multiple studies have examined the change in galectin levels after chemotherapy in breast cancer. For example, galectin-1 levels in breast cancer have been shown to increase after treatment that included chemotherapy, hormonal therapy, and immunotherapy [46]. The differences between these findings and what we observed in our NSCLC study may reflect the utilization of hormonal therapy for breast cancer, which does not have a clinical role in the treatment of lung cancer. Furthermore, it is possible that there are differences in galectin levels based on the chemotherapeutic agents used, or differences in the cancer biology itself. It is of note that in our study there was a trend towards decreased galectin-1 levels after exposure to chemotherapy. Furthermore, another breast cancer study demonstrated that there was no change in galectin-3 levels in response to doxorubicin [47]. Doxorubicin is not routinely utilized in the treatment of lung cancer; therefore, this may explain why we observed a difference in galectin-3 levels in our study.

Additionally, we found that there were no changes in galectin levels observed with other treatment modalities. Previously, radiation treatment of Lewis lung carcinoma in mice has demonstrated an increase in galectin-1 secretion [48]. However, radiation treatment resulting in an increase in galectin-1 levels was not seen in Figure 3 and may be because our patient population had more stage I and II disease compared to the general lung cancer population. Thus, the tumor burden in our patients may not have been sufficient to elicit increased galectin-1 levels despite irradiation, in comparison to the mice examined in the study mentioned above. It is also possible that there is no difference in adenocarcinoma and squamous cell carcinoma, as examined in our study, compared to Lewis lung carcinoma. These findings regarding treatment and galectin levels are important because, to our knowledge, no study has explored galectin levels after all treatment types in lung cancer.

Although the patients that had lower galectin-1 levels were more likely to have previous metastatic disease, serum galectin-1, -3, and -9 levels did not predict future metastasis. Earlier studies have demonstrated that elevated levels detected by ELISA or increased expression of galectin-3 detected by IHC correlate with metastasis [24,34]. We hypothesize that the observed differences are due to our patient population. Only 39 patients (45%) in our population had combined stage III (n = 18) or stage IV (n = 21) disease, and the national average for combined stage III and IV disease is 75% [31]. Therefore, our study population could be viewed as healthier than the national average lung cancer population. This observation may also explain the low metastasis rate we observed in our study, with only 10 patients developing metastatic disease. Furthermore, since our patient population primarily consisted of surgically resectable cases, there is a mild specimen bias. This bias arises from planned resections, which involve the preparation for tissue storage. In contrast, needle biopsies, often performed during in-office procedures, may not be captured in the same way. Specifically, out of the 87 samples obtained, our study includes only four samples taken from lymph nodes (n = 2) or chest wall (n = 2) biopsies. Finally, given that our population had a significant proportion of surgically resectable diseases, a higher number of patients were cured compared to the national average.

Abnormal galectin-1 levels correlated with decreased overall survival, as depicted in Figure 6B with no difference for galectins-3 and -9 in Figure 6C and Figure 6D, respectively. The lack of differences observed for galectin-3 levels and the differences observed for galectin-1 levels correlate with previous studies [22,34,49]. It is of note that there was no difference in OS when galectin-1 levels were separated into low, normal, and high values, as seen in Figure 6A, albeit it approached significance (*p* = 0.0953). This finding correlates with our earlier discovery that individuals with metastatic disease exhibited lower galectin-1 values at the time of sample procurement.

Subsequent analysis highlighted that when patients were grouped by individual histologic subtypes there was no difference in OS. As there is a separation in the Kaplan–Meier curves in Figure 7 and a difference observed in the NSCLC population, the lack of difference in this subgroup analysis is likely due to smaller sample sizes. Nevertheless, individuals with surgically resectable NSCLC retained a survival difference, as depicted in Figure 8B. This suggests that galectin-1 assessed for curative surgical resection of NSCLC may serve as a prognostic factor for OS. Further prospective trials are needed to confirm this finding.

However, when demographic factors and NSCLC stage were adjusted for in a Cox proportional-hazards model, abnormal galectin-1 levels were not significantly associated with decreased OS, yet with borderline significance (*p* = 0.0513), which is possibly due to our relatively small sample size. Validation of these findings through prospective clinical studies is warranted as prognostic biomarkers in oncology are critical for guiding treatment decisions and informing patients on prognosis. This is particularly crucial in non-small cell lung cancer (NSCLC), where there exists a time window for initiating adjuvant chemotherapy [50].

The mechanism by which OS is impacted by abnormal galectin-1 expression is not fully elucidated. One trial has demonstrated that galectin-1 attenuated tumor response to radiation, and that, by inhibiting galectin-1 in addition to radiation treatment, there was a decreased tumor growth and dissemination [48]. Thus, the patients with normal galectin-1 levels may represent a favorable genetic phenotype that corresponds to improved OS, as seen in our study. This speculation is further supported by our previous research demonstrating that when *KIT* or *PTEN* cancer-critical genes are mutated in lung cancer, galectin-1 levels are elevated [27]. However, further research into the mechanism by which abnormal galectin-1 correlates with OS is necessary.

### 4.2. Limitations

While our study aims to mitigate the influence of confounding variables on galectin-1, -3, and -9 levels by examining patients’ demographic factors and cancer stage, we have not accounted for other potential factors that may affect galectin levels, such as comorbidities, lifestyle factors, or genetic predispositions. Additionally, other factors such as socioeconomic status and patients’ living conditions may influence galectin levels that are not easily quantifiable by chart review.

Additionally, our sample size is small, with only 87 total patients. Although it is homogenous for those with the most common types of NSCLC, larger studies are necessary to validate our findings. Furthermore, our sample size included 18 patients with stage III disease and 21 patients with stage IV disease, giving a combined total of 45% with advanced disease. This number does not represent the general population as >75% of lung cancer patients have either stage III or stage IV disease [31]. Therefore, our patient population is not as homogenous across all stages of disease, as seen by our low numbers of stage IIIB and stage IIIC disease. This imitation of our study makes it less generalizable to patients with later stage disease, and more generalizable to patients with early-stage disease.

Although we could assess galectin levels regardless of previous treatments received, serum was only taken once during the day of tissue sample collection, and serial galectin levels could not be obtained. This limits the ability to monitor how different lung cancer treatments would impact future galectin level changes or if changes in galectin levels correlate with survival. The relationship between galectin levels after treatment is of interest in oncology, as chemotherapy-induced galectin-3 increases have been correlated with significantly lower rates of recurrence in breast cancer [51].

In our study, we have analyzed galectin-1, galectin-3, and galectin-9, as these are among the most extensively studied galectins in the context of lung cancer [16]. Given the complexity of lung cancer’s molecular biology, it may be of interest to analyze additional galectins, as well as other relevant lung cancer biomarkers such as ALK, TP53, and EGFR, simultaneously, to gain deeper insights into their implications in lung cancer. This may lead to a mechanistic understanding of how these biomarkers reflect cancer biology, which is not represented in our study, but remains incredibly important for understanding lung cancer and developing novel approaches to prognose and treat it. Additionally, it may provide an explanation for why galectin-3 is elevated in adenocarcinoma compared to other types of lung cancer.

Lastly, our study is exploratory in nature and of retrospective design. Although useful for identifying trends and hypothesis generation, this limits the strength of our findings. Further prospective studies are necessary to validate the results of this investigation to better assess the utility of galectins in the prognosis of NSCLC.

## 5. Conclusions

Our research builds upon previous studies examining the alterations in galectin-3 levels in lung cancer. It is the first study to demonstrate that galectin-3 levels are elevated in adenocarcinoma patients compared to patients with squamous cell carcinoma, which may have future treatment implications with the development of galectin-3 inhibitors.

Assessment of whether galectin levels are affected by all lung cancer treatment types has not been previously performed. Although we found that galectin-3 levels were lower in patients treated with chemotherapy, confirming our findings requires prospective studies with serial testing to assess the impact on treatment intervention.

Finally, lower galectin-1 levels correlated with previous metastatic disease, and abnormal galectin-1 levels correlated with decreased overall survival in NSCLC. Furthermore, the significant difference in OS remained in patients that had surgically resectable NSCLC but not in patients with unresectable disease. This finding highlights the potential utility of galectin-1 as a prognostic biomarker for resectable NSCLC. We did not demonstrate significant OS changes in multivariable analysis with galectin-1 levels; however, they approached statistical significance. Future prospective studies are necessary to assess the utility of galectin-1 as a prognostic marker for surgically resectable NSCLC, since it is crucial to have biomarkers that guide prognosis and inform treatment decisions in this population.

## Figures and Tables

**Table 1:** Galectin-1, -3, and -9 levels compared by patient demographics.

Characteristics	Galectin-1: Mean (SD), *n*	Galectin-3: Mean (SD), *n*	Galectin-9: Mean (SD), *n*
Age	*n* = 87	*n* = 84	*n* = 87
	3.088 (0.3980), *n* = 87	2.463 (0.5432), *n* = 84	2.204 (0.4171), *n* = 87
*p*-value	0.0850	0.3580	0.3972
Sex	*n* = 86	*n* = 83	*n* = 86
Female	3.149 (0.0665), *n* = 36	2.595 (0.0939), *n* = 33	2.321 (0.0682), *n* = 36
Male	3.043 (0.0564), *n* = 50	2.377 (0.0763), *n* = 50	2.117 (0.0579), *n* = 50
*p*-value	0.1140	0.0377	0.0124
Race	*n* = 87	*n* = 84	*n* = 87
Black, African American	3.014 (0.1016), *n* = 15	2.491 (0.1470), *n* = 14	2.075 (0.1068), *n* = 15
Multicultural Cohort	2.730 (0.2279), *n* = 3	2.355 (0.3172), *n* = 3	2.139 (0.2410), *n* = 3
White	3.120 (0.0474), *n* = 69	2.451 (0.0569), *n* = 67	2.234 (0.0498), *n* = 69
*p*-value	0.1846	0.9261	0.3949
Ethnicity	(*n* = 87)	(*n* = 84)	(*n* = 87)
Non-Spanish, Non-Hispanic	3.102 (0.0423), *n* = 85	2.461 (0.0603), *n* = 82	2.212 (0.0451)), *n* = 85
Spanish, Hispanic	2.516 (0.2760), *n* = 2	2.523 (0.3863), *n* = 2	1.833 (0.2938), *n* = 2
*p*-value	0.0195	0.5628	0.1029
Smoking Status	(*n* = 87)	(*n* = 84)	(*n* = 87)
Current Smoker	2.998 (0.0765), *n* = 27	2.442 (0.1049), *n* = 27	2.094 (0.0785), *n* = 27
Never Smoker	3.199 (0.1988), *n* = 4	2.835 (0.3147), *n* = 3	2.603 (0.2039), *n* = 4
Previous Smoker	3.124 (0.0531), *n* = 56	2.452 (0.0742), *n* = 54	2.228 (0.0545), *n* = 56
*p*-value	0.3481	0.4684	0.0553
BMI	(*n* = 81)	(*n* = 78)	(*n* = 81)
Underweight	2.919 (0.2394), *n* = 3	2.221 (0.3260), *n* = 3	2.003 (0.2473), *n* = 3
Healthy	3.090 (0.0784), *n* = 28	2.451 (0.1087), *n* = 27	2.125 (0.0810), *n* = 28
Overweight	3.065 (0.0701), *n* = 35	2.435 (0.0983), *n* = 33	2.213 (0.0724, *n* = 35
Obese	3.201 (0.1071), *n* = 15	2.502 (0.1458), *n* = 15	2.327 (0.1106), *n* = 15
*p*-value	0.6376	0.8879	0.4216

Mean, standard deviation (SD), and *p*-values of serum galectins by patient demographics. Galectin values are represented in log10. Age was measured numerically in years with a mean age of 65.89. Hawaiian/Pacific Islander, Latino, and Multi-racial were combined and called Multicultural Cohort. Body Mass Index (BMI) includes underweight (<18.5), healthy (18.5–24.9), overweight (25–29.9), and obese (>29.9). Bivariate fit analysis was performed on age. A two-tailed *t*-test was used for two group comparisons: sex and ethnicity. One-way ANOVA was utilized for multiple group comparisons: race and smoking status.

**Table 2:** Galectin-1, -3, and -9 levels compared by previous lung cancer treatment types.

	Radiation	Surgery	Chemotherapy	Targeted Therapy	Immunotherapy
Galectin-1 Mean (SD)	*n* = 84	*n* = 84	*n* = 83	*n* = 81	*n* = 82
No Treatment	3.107 (0.0450), *n* = 66	3.060 (0.1633), *n* = 6	3.127 (0.0532), *n* = 56	3.078 (0.0443), *n* = 78	3.096 (0.0448), *n* = 79
Treatment	3.041 (0.0957), *n* = 18	3.086 (0.0453), *n* = 78	2.998 (0.0765), *n* = 27	3.200 (0.2258), *n* = 3	2.988 (0.2301), *n* = 3
*T*-test *p* value	0.2703	0.5590	0.0852	0.7010	0.3223
Galectin-3 Mean (SD)	*n* = 81	*n* = 81	*n* = 80	*n* = 78	*n* = 79
No Treatment	2.485 (0.0680), *n* = 64	2.146 (0.2210), *n* = 6	2.523 (0.0735), *n* = 54	2.447 (0.0634), *n* = 75	2.465 (0.0626), *n* = 76
Treatment	2.318 (0.1320), *n* = 17	2.470 (0.0625), *n* = 75	2.290 (0.1060), *n* = 26	2.637 (0.3168), *n* = 3	2.256 (0.3151), *n* = 3
*T*-test *p* value	0.1324	0.9186	0.0375	0.721	0.2579
Galectin-9 Mean (SD)	*n* = 84	*n* = 84	*n* = 83	*n* = 81	*n* = 82
No Treatment	2.208 (0.0521), *n* = 66	2.282 (0.1729), *n* = 6	2.249 (0.0563), *n* = 56	2.217 (0.0471), *n* = 78	2.229 (0.0466), *n* = 79
Treatment	2.204 (0.0997), *n* = 18	2.204 (0.0480), *n* = 78	2.118 (0.0810), *n* = 27	1.991 (0.2401), *n* = 3	1.856 (0.2393), *n* = 3
*T*-test *p* value	0.4867	0.3322	0.0940	0.1797	0.0649

Means, standard deviations (SD), and *p*-values of serum galectins by previous lung cancer treatment type. Galectin levels are represented in log10. A two-tailed *t*-test was utilized for two group comparisons for each treatment type.

## Data Availability

All data from the Prisma Health Biorepository supporting this study’s findings are available in the Supplemental Data File titled S1, Investigational Information, of this article. As patient HIPPA protected information, such as dates, were utilized to determine overall survival and time to metastasis, personal identifiable information is not available to individuals not covered by IRB approval.

## Supplementary Materials

The following supporting information can be downloaded at https://www.mdpi.com/article/10.3390/cancers16061165/s1, Data File S1: Investigational Information. Data File S2: Hazard Ratios.
